# Effects of cardiac pacemakers on left ventricular volumes and function assessed by 3D echocardiography, Doppler method, and global longitudinal strain

**DOI:** 10.1186/s43044-021-00138-9

**Published:** 2021-02-22

**Authors:** Moustafa Dawood, Eman Elsharkawy, Mohamed Ayman Abdel-Hay, Moustafa Nawar

**Affiliations:** Alexandria Faculty of Medicine, Alexandria, Egypt

**Keywords:** Cardiac output, 3D echocardiography, Global longitudinal strain, Single chamber, Dual chamber cardiac pacemakers, Pacing hemodynamics

## Abstract

**Background:**

Many previous studies reported the negative effects of right ventricular (RV) pacing on the left ventricular (LV) structure and ejection fraction. Studying pacing hemodynamics is essential to understand these detrimental effects. In this study, we tried to understand RV pacing effects on LV volumes and function using advanced tools like 3D echo and global longitudinal strain (GLS). This was a prospective study of 175 consecutive patients (LVEF>50%) presented permanent pacing. Of 175 patients, only 50 patients met study criteria, divided into two groups (single or dual pacing). LV volumes and function were assessed by full-volume 3D echocardiography and GLS before pacing, at 1-week and 6-month post-pacing. Cardiac output (COP) was calculated by pulsed wave Doppler method and 3D echo.

**Results:**

Doppler method results were similar to 3D echo in calculating SV and COP. At 1-week post pacing, both groups showed a significant decrease in SV due to a drop in EDV while ESV did not change significantly. Despite the drop in SV, there was a significant increase in cardiac output (COP) due to achieving higher heart rates post-pacing. There was a significant drop in EF and GLS in both groups.

At 6 months, SV continued to decrease with a corresponding decrease in COP and LVEF. This drop in SV was due to a significant increase in ESV while EDV did not show a significant change at a 6-month follow-up. Also, the drop EF and GLS became more significant.

There were no significant differences between both groups regarding the changes in LV volumes (EDV, ESV, SV), LVEF or GLS throughout the study (pre-pacing, at 1-week and 6-months post pacing). However, dual-chamber pacing group provided higher heart rates and as a result higher COP than the single-chamber group.

**Conclusions:**

RV pacing led to a significant drop in LV COP, ejection fraction (EF), and GLS over short- and long-term duration. Dual chamber pacing provided higher COP than a single chamber pacing. This was due to tracking the S. A node with pacing at higher heart rates not due to an increase in SV and preserving atrioventricular synchrony. Both Doppler method and 3D echo can be used to calculate SV and COP.

**Supplementary Information:**

The online version contains supplementary material available at 10.1186/s43044-021-00138-9.

## Background

Cardiac pacing is the established treatment of the heart block since more than 50 years [1]. At first, pacemakers were capable of pacing only one chamber of the heart, usually the right ventricle. With more advances, dual-chamber pacemakers were developed to sense and pace, both the atrium and the ventricle and thus are able to achieve atrioventricular synchrony [2]. A single-chamber pacemaker has only one lead implanted (usually in the right ventricle) so it controls the activity of the ventricles regardless the condition of the atria whether in systole or in diastole which means that the atria may contract against closed AV valves while the ventricles are in systole causing pacemaker syndrome (dyspnea and symptoms of pulmonary congestion) [3]. However, a dual chamber has two leads, one in the right atrium and one in the right ventricle resembles the normal activities of the heart and reflects intrinsic depolarization. It gives time to the atria to empty in the ventricles preserving the atrial kick and was quickly classified as physiologic pacing mode [2].

As a result, dual-chamber pacing, as compared with single-chamber ventricular pacing, improves hemodynamic function [4–6], but the clinical benefit is uncertain. Many studies suggest that dual-chamber pacing has better outcomes regarding symptoms, exercise tolerance, and ambulatory blood pressure monitoring. It was associated with a lower incidence of atrial fibrillation, stroke, and heart failure than single-chamber pacing [7]. There was also evidence of improved survival [8]. Some current guidelines recommend dual-chamber pacing except in patients with atrial flutter or fibrillation [9].

On the other hand, a number of randomized studies suggested no significant differences between single-chamber and dual-chamber pacing in the rates of atrial fibrillation, heart failure, or a composite of stroke, ischemic attack or death [10–12]. In this study, we aimed to study pacing hemodynamics, effects on left ventricular (LV) volumes, and function over a 6-month interval. We used the standard Doppler wave methods in addition to 3D echo to calculate SV and COP. Being more superior to 2D echo, the remaining LV volumes and ejection fraction were calculated using 3D echocardiography.

## Methods

The study was approved by our faculty of medicine ethics committee. All patients provided written informed consent.

### Patients’ selection

Patient recruitment started from October 2017 to August 2018. During this period, 175 consecutive patients presented to our university hospitals for device implantation.

The exclusion criteria included the presence of more than mild valvular heart disease, left ventricular ejection fraction less than 50%, presence of ischemic heart disease, and recent cardiac surgery during the last 3 months before enrollment. Patients with poor echo windows, patients with slow atrial fibrillation or other types of arrhythmia that can affect stroke volume measurement, debilitated or cancer patients with expected survival less than 1 year and patients with previously implanted devices were excluded. From 175 patients, 124 patients were excluded. Sixty-five patients were presented with reduced LV systolic function for cardiac resynchronization therapy. Sixteen patients were assigned for ICD devices as a primary or secondary prevention with no indication for permanent pacing. Twenty-two patients had previously implanted devices at elective replacement period and were scheduled for battery replacement. Seven patients were subjected to reoperation due to device related complications (4 with twiddler syndrome and 3 for device extraction due to bloodstream or device-related infection). Eight patients presented with slow atrial fibrillation or SSS. Two patients had poor echo views, and 4 patients had significant valvular heart disease. One patient died before the second follow-up. The remaining 50 patients were distributed to have single chamber (27 patients) or dual chamber pacemaker (23 patients).

### Patient characteristics

Patient’s demographic data and indications for pacing were collected and revised 24 h before pacing. Patient’s age ranged from 12 to 97 years with mean age of 63.12 ± 16.85 years. According to gender, 27 patients were males (54%) and 23 were females (46%). All the patients had structurally normal heart confirmed by echocardiography (Table 1).

**Table 1:** Summary of patients’ characteristics

Predisposing factors	Total (***n***=50)	
	No.	%
**Age** (years)	63.12 ± 16.85	
Mean ± SD.		
**Sex**		
Male	27	54.0
Female	23	46.0
**DM**	13	26.0
**HTN**	21	42.0

### Data collection: 2D echocardiography and PW Doppler method

A full 2D echocardiographic study was done to exclude patients with significant valvular heart disease, IHD, or reduced LVEF. The baseline heart rate was recorded. Our study used two different methods to calculated SV; full volume 3D Echo and Doppler method by calculating VTI at LVOT by PW Doppler. Both methods were applied before pacing and at follow-up visits. In order to avoid any confounding factors during SV and COP estimation, pre-pacing LVOT diameter was recorded and reused at follow-up for each patient. Also, to avoid confounding factors between both methods during COP calculation, the same heart rate was multiplied by 3D and Doppler SV at every visit.

### Full-volume 3D Echo acquisition and GLS analysis

A 3D full-volume acquisition of the left ventricle was done using the Philips Medical iE33 echocardiography system with X5-1 transthoracic probe. For 3D full-volume acquisitions, ensuring adequate frame rate and packet size and capture of the full cardiac cycle, the system obtained a 30°–30° pyramid over 4 to 6 alternate gated cardiac cycles. The resulting dynamic 3D full volume sector was reviewed and navigated through immediately to ensure all areas of interest have been captured. All the acquisitions were ECG gated and patients were told to hold their breath during acquisition to avoid stitch artifacts. After completion of the study, 3D acquired data were transferred to Q-lab 10 for off-line analysis. In order to calculate LV volumes and EF, both the long and short LV axes were adjusted to get maximum LV dimensions and to avoid foreshortening then five landmarks were chosen to initiate edge detection by semi-automated quantification software. Four landmarks were placed at mitral annulus, and the fifth was placed at the apex in apical four or apical two views. The software delineated LV boundaries automatically, but it allowed manual modifications to include or exclude any part for more accurate adjustment of LV borders. After borders delineation, the software automatically calculated EDV, ESV, SV, COP, and EF.

For GLS calculation, 2D gated acquisition of the apical views (apical four, two, and three) were done according to the standard techniques. Patients were told to hold their breath during acquisition, and foreshortening was avoided. GLS was calculated for each of the three apical view, and then, mean GLS was calculated automatically.

### Pacemaker implantation and programming

Implantation was performed according to the operator’s preference. Being easily accessible, more stable and non-inferior to RV septal pacing [13, 14], RV apex was selected as the site of RV lead implantation in all candidates. Patients with single-chamber pacemakers were programmed to VVIR mode. Patients with dual-chamber pacing were programmed to DDDR mode. Rate responsive mode was selected as it resulted in better outcomes regarding patient quality of life and exercise tolerance [15]. Suggested settings for dual-chamber pacemakers were lower and upper rate limits of 60 beats per minute and 130 beats per minute, respectively. For single-chamber pacemakers, the suggested lower and upper rate limits were 60 beats per minute and 130 beats per minute, respectively. In order to maximize the contribution of the atrial kick to SV in the DDDR group, dynamic AV time delay [16] was selected with resting paced/sensed AV time delay adjusted to 200/150 ms [16, 17]. Patients were recruited 7–10 days post-pacing and after 6 months. Device interrogation was done to check the adjusted pacing parameters and acquire ventricular pacing percentage. Also, 3D echo and GLS were calculated at follow-up visits as described before.

### Statistical analysis

The database was maintained and analyzed by an independent data-management group. To assess the distribution of the data derived from this study, we calculated the standardized skewness and kurtosis of each of the variables. Normally distributed values were expressed as mean and skewed values as the median (interquartile range). Paired two-tailed group comparisons were made with Student’s *t* (parametric) or Wilcoxon signed-rank (non-parametric) tests as appropriate. *p* values of less than 0.05 were regarded as significant.

## Results

It was found that Doppler method was not inferior to 3D echo in calculating SV and COP (Table 2). There was no significant difference between both methods in both groups pre-pacing or at follow-up visits. Many studies compared 2D and 3D Echo to CMR as being the gold standard method. This was the first study to compare 3D echo to Doppler method for COP calculation.

**Table 2:** Comparison between 3D and PW methods according to COP in each group

COP	3D	PW	^**W**^_**X**_	***P***
**Single chamber**				
Post-pacing (7–10 days)	3.69 ± 1.13	3.68 ± 1.11	0.141	0.888
Follow up after 6 months	3.27 ± 1.12	3.40 ± 1.03	1.830	0.067
**Dual chamber**				
Post-pacing (7–10 days)	4.48 ± 1.14	4.35 ± 1.04	1.511	0.131
Follow up after 6 months	3.96 ± 1.04	3.90 ± 0.83	0.070	0.944

WX, p: Wx and *P* values for Wilcoxon signed-rank test for comparing between single and dual chamber

**Fig. 1:**
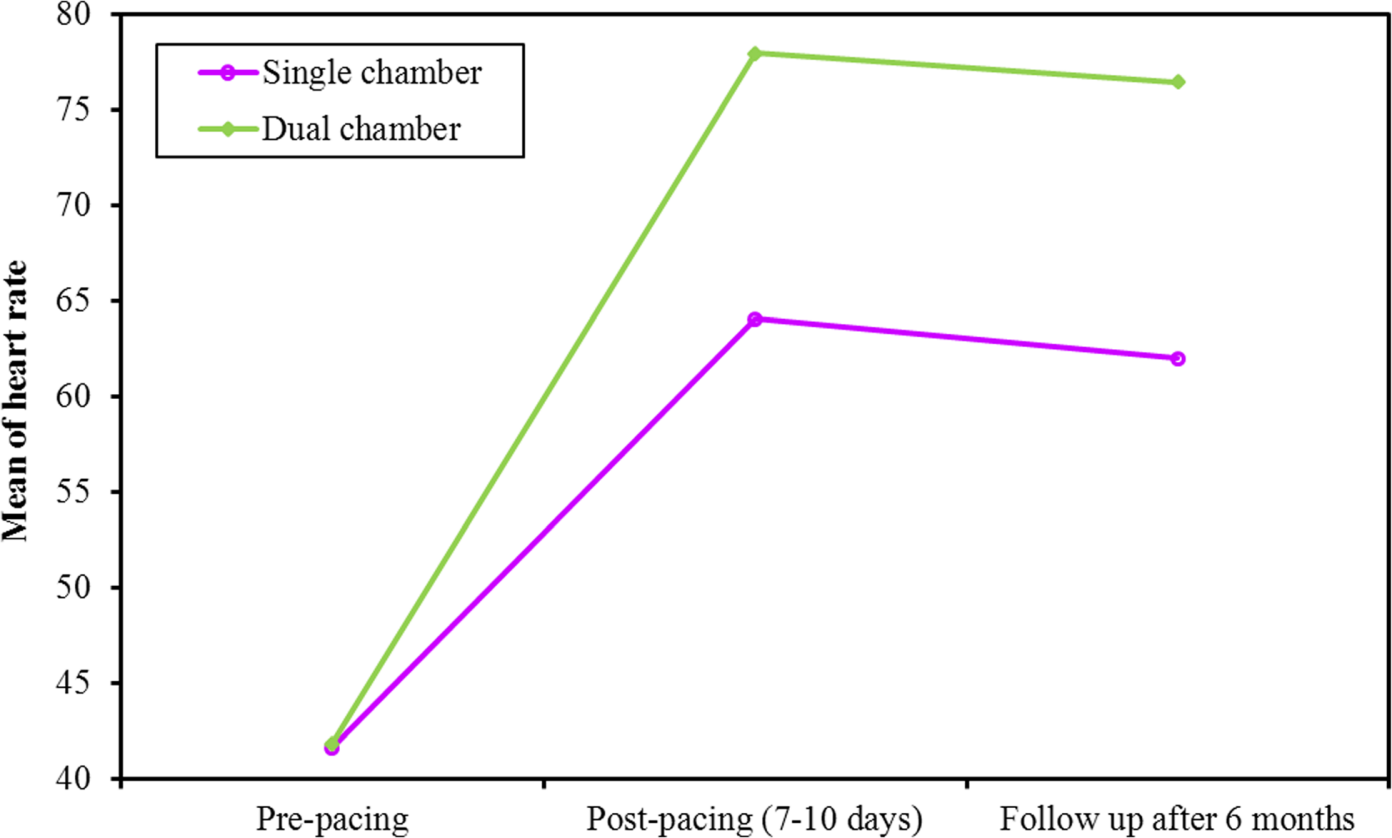
Comparison between the different periods according to heart rate in each group

**Fig. 2:**
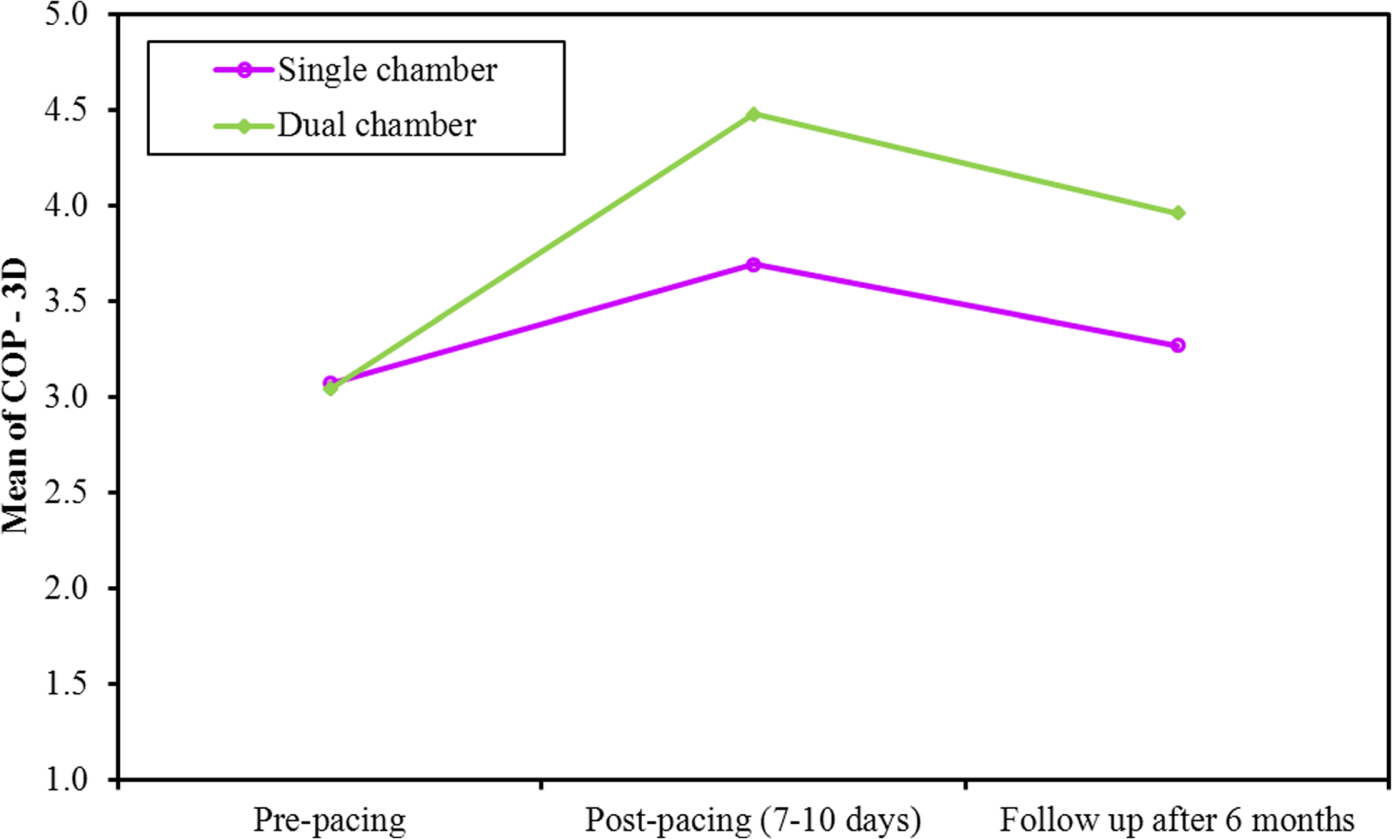
Comparison between the different periods according to 3D COP in each group

Device interrogation showed high-ventricular pacing percentage at 6-month follow-up. All the patients were pacemaker dependent with mean VP% of 92±3%. There were no significant differences between both groups before implantation regarding these parameters (ESV, EDV, SV, HR, COP, EF, and GLS). Both HR and COP showed a significant increase post-pacing. However, at post-pacing visits, dual-chamber group achieved higher HR by tracing the patients’ intrinsic heart rate (*p* value < 0.001). COP is the result of multiplying SV by HR. Consequently, dual-chamber pacemakers provided higher COP than single-chamber devices (*p* value < 0.032, < 0.026 at 1-week and 6 months, respectively, Figs. 1 and 2). There were no significant differences between both groups in EDV, ESV, SV, EF, and GLS at 1-week or 6 months post-pacing (Table 3).

**Table 3:** Comparison between single- and dual-chamber pacing groups including all the measured parameters at the three time intervals (pre-pacing, at 1 week and 6 months post pacing)

Measurement	Pre-pacing	*p* value	Post pacing at 1 week	*p* value	Post pacing At 6 months	*p* value
**EDV** (ml)						
Single-chamber	102.6 ± 25.95	0.360	86.81 ± 25.09	0.259	88.93 ± 30.39	0.098
Dual-chamber	109.0 ± 22.91		93.43 ± 18.80		97.04 ± 19.62	
**ESV** (ml)						
Single-chamber	28.48 ± 9.67	0.055	28.96 ± 11.25	0.080	36.52 ± 21.87	0.143
Dual-chamber	34.43 ± 11.75		34.91 ± 12.24		44.65 ± 15.66	
**SV** (ml)						
Single-chamber	74.07 ± 20.20	0.944	57.78 ± 17.47	0.880	52.56 ± 17.54	0.962
Dual-chamber	73.7 ± 17.35		58.52 ± 17.02		52.35 ± 13.13	
**HR** (bpm)						
Single-chamber	41.63 ± 8.45	0.938	64.04 ± 9.79	<0.001^*^	62.00 ± 6.45	<0.001^*^
Dual-chamber	41.83 ± 9.36		77.96 ± 10.85		76.43 ± 9.43	
**COP** (L/m)						
Single-chamber	3.07 ± 0.99	0.853	3.69 ± 1.13	0.032^*^	3.27 ± 1.12	0.026^*^
Dual-chamber	3.04 ± 0.90		4.48 ± 1.14		3.96 ± 1.04	
**EF** (%)						
Single-chamber	72.41 ± 6.55	0.156	65.96 ± 8.41	0.058	59.56 ± 9.85	0.071
Dual-chamber	69.35 ± 8.46		60.74 ± 10.62		54.35 ± 10.04	
**GLS** (%)						
Single-chamber	-19.41 ± 3.53	0.819	-15.44 ± 4.09	0.446	-13.52 ± 4.66	0.766
Dual-chamber	-19.65 ± 3.98		-14.52 ± 4.39		-13.13 ± 4.45	

Values are presented as mean ± SD

*Statistically significant at *P* ≤ 0.05

### Pacing effects on LV volumes and function

**Fig. 3:**
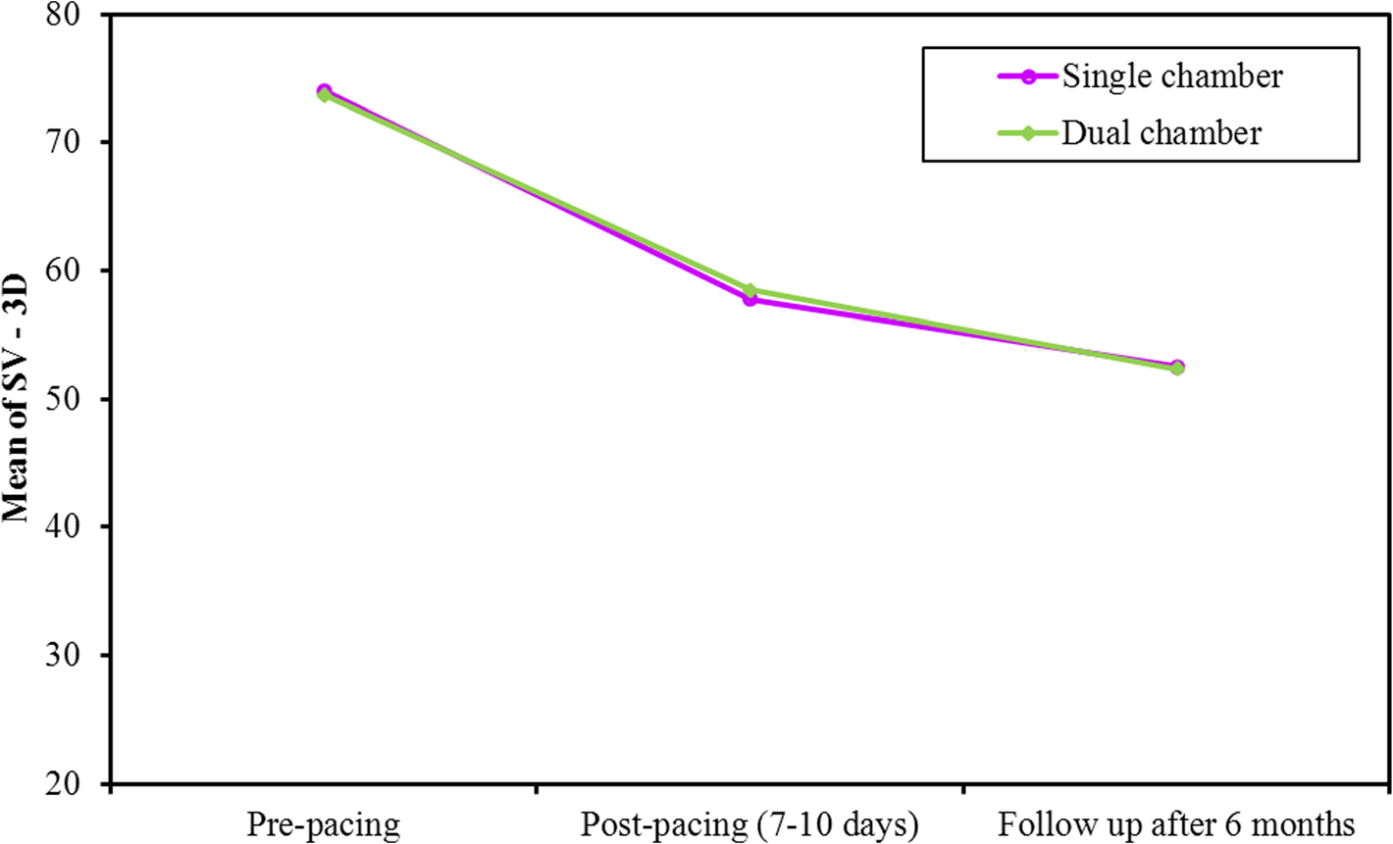
Comparison between the different periods according to 3D SV in each group

**Fig. 4:**
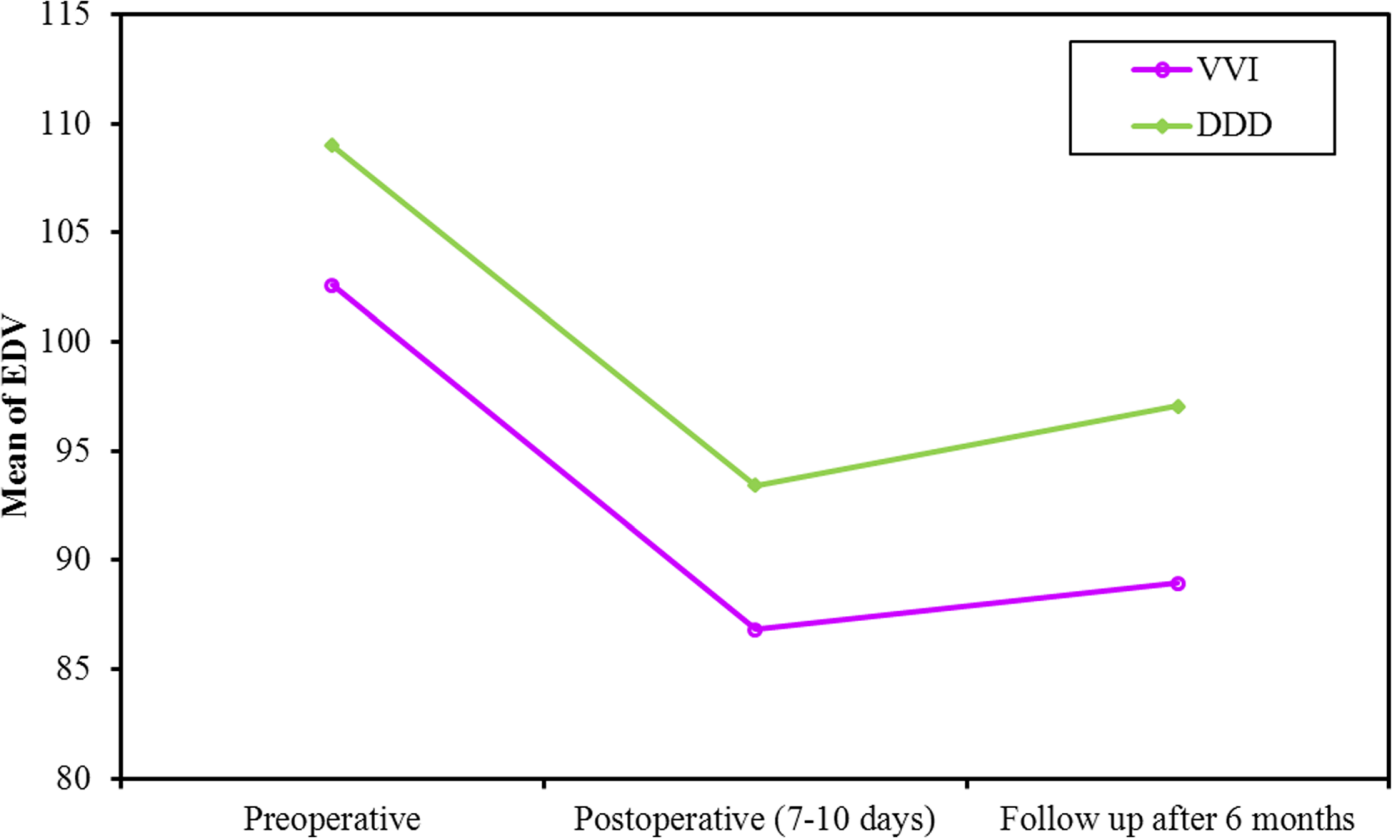
Comparison between the different periods according to EDV in each group

**Fig. 5:**
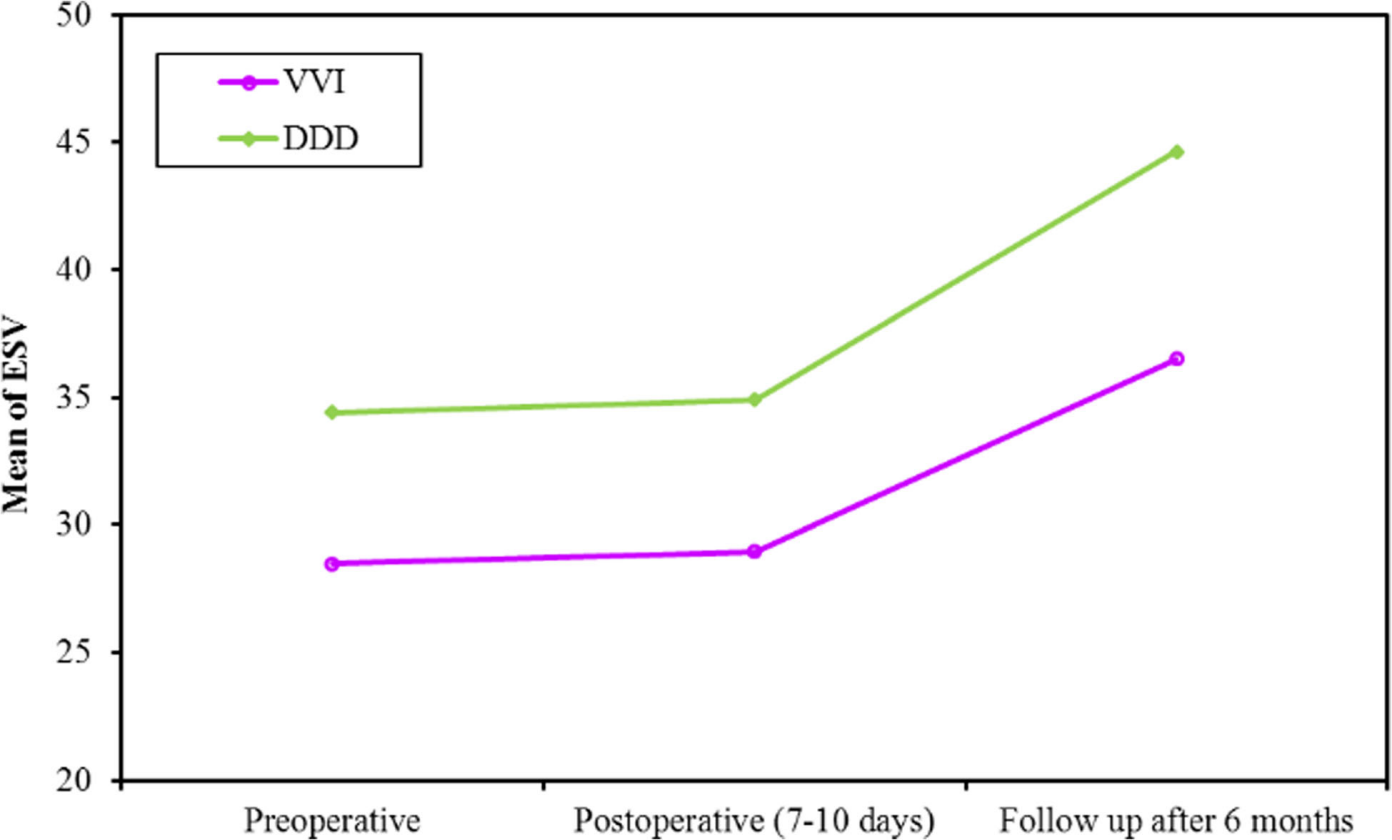
Comparison between the different periods according to ESV in each group

The study population showed a continuous reduction in SV, EF, and GLS over short- and long-term periods. At 1-week post pacing, both groups showed a significant reduction in SV which continued to decrease at 6 months (*p* value < 0.001, <0.001, respectively). Also, the decrease in SV from 1 week to 6 months was significant in single and dual chamber groups. (*p* value = 0.018, 0.048, respectively, Fig. 3). At 1 week, SV decreased due to acute reduction in EDV while ESV did not change significantly. At 6 months, SV decreased due to delayed reduction in ESV while EDV did not change significantly (Figs. 4 and 5, Table 4).

**Table 4:** Pacing related changes in all the measured parameters at the three time intervals (pre-pacing, at 1 week and 6 months post pacing)

Measurement	Pre-pacing	Post pacing at 1 week	Post pacing at 6 months	Test	*P* value
**EDV** (ml)				Fr	
Single-chamber	102.6 ± 25.95	86.81 ± 25.09	88.93 ± 30.39	30.882^*^	<0.001^*^
Dual-chamber	109.0 ± 22.91	93.43 ± 18.80	97.04 ± 19.62	19.089^*^	<0.001^*^
**Sig. bet. Periods**					
Single-chamber	p_1_<0.001^*^, p_2_=0.002^*^, p_3_=0.338				
Dual-chamber	p_1_<0.001^*^, p_2_<0.001^*^, p_3_=0.307				
**ESV** (ml)				F	
Single-chamber	28.48 ± 9.67	28.96 ± 11.25	36.52 ± 21.87	4.411^*^	0.017^*^
Dual-chamber	34.43 ± 11.75	34.91 ± 12.24	44.65 ± 15.66	8.077^*^	0.001^*^
**Sig. bet. Periods**					
Single-chamber	p_1_=1.000 , p_2_=0.175, p_3_=0.040^*^				
Dual-chamber	p_1_=1.000 , p_2_=0.018^*^, p_3_=0.004^*^				
**SV** (ml)				F	
Single-chamber	74.07 ± 20.20	57.78 ± 17.47	52.56 ± 17.54	40.039^*^	<0.001^*^
Dual-chamber	73.7 ± 17.35	58.52 ± 17.02	52.35 ± 13.13	33.559^*^	<0.001^*^
**Sig. bet. Periods**					
Single-chamber	p_1_<0.001^*^,p_2_<0.001^*^,p_3_=0.018^*^				
Dual-chamber	p_1_<0.001^*^,p_2_<0.001^*^,p_3_=0.048^*^				
**HR** (bpm)				F	
Single-chamber	41.63 ± 8.45	64.04 ± 9.79	62.00 ± 6.45	186.913^*^	<0.001^*^
Dual-chamber	41.83 ± 9.36	77.96 ± 10.85	76.43 ± 9.43	107.586^*^	<0.001^*^
**Sig. bet. Periods**					
Single-chamber	p_1_<0.001^*^, p_2_<0.001^*^, p_3_=0.325				
Dual-chamber	p_1_<0.001^*^, p_2_<0.001^*^, p_3_=1.000				
**COP** (L/m)				Fr	
Single-chamber	3.07 ± 0.99	3.69 ± 1.13	3.27 ± 1.12	16.020^*^	<0.001^*^
Dual-chamber	3.04 ± 0.90	4.48 ± 1.14	3.96 ± 1.04	30.776^*^	<0.001^*^
**Sig. bet. Periods**					
Single-chamber	p_1_<0.001^*^,p_2_=0.247,p_3_=0.010^*^				
Dual-chamber	p_1_<0.001^*^,p_2_=0.002^*^,p_3_=0.027^*^				
**EF** (%)				F	
Single-chamber	72.41 ± 6.55	65.96 ± 8.41	59.56 ± 9.85	28.566^*^	<0.001^*^
Dual-chamber	69.35 ± 8.46	60.74 ± 10.62	54.35 ± 10.04	26.749^*^	<0.001^*^
**Sig. bet. periods**					
Single-chamber	p_1_<0.001^*^,p_2_<0.001^*^,p_3_=0.001^*^				
Dual-chamber	p_1_<0.001^*^,p_2_<0.001^*^,p_3_=0.011^*^				
**GLS** (%)				F	
Single-chamber	-19.41 ± 3.53	-15.44 ± 4.09	-13.52 ± 4.66	62.924^*^	<0.001^*^
Dual-chamber	-19.65 ± 3.98	-14.52 ± 4.39	-13.13 ± 4.45	50.749^*^	<0.001^*^
**Sig. bet. periods**					
Single-chamber	p_1_<0.001^*^, p_2_<0.001^*^, p_3_<0.001^*^				
Dual-chamber	p_1_<0.001^*^, p_2_<0.001^*^, p_3_=0.001^*^				

Fr: Friedman test, sig. bet. periods was done using post hoc test (Dunn’s)

F: *F* test (**ANOVA**) with repeated measures

p: *P* value for post hoc test (Bonferroni) for comparison between different periods

p_1_: *P* value for comparing between preoperative and post-operative (7–10 days)

p_2_: *P* value for comparing between preoperative and follow-up after 6 months

p_3_: *P* value for comparing between post-operative (7–10 days) and follow-up after 6 months

^*^Statistically significant at *P* ≤ 0.05

**Fig. 6:**
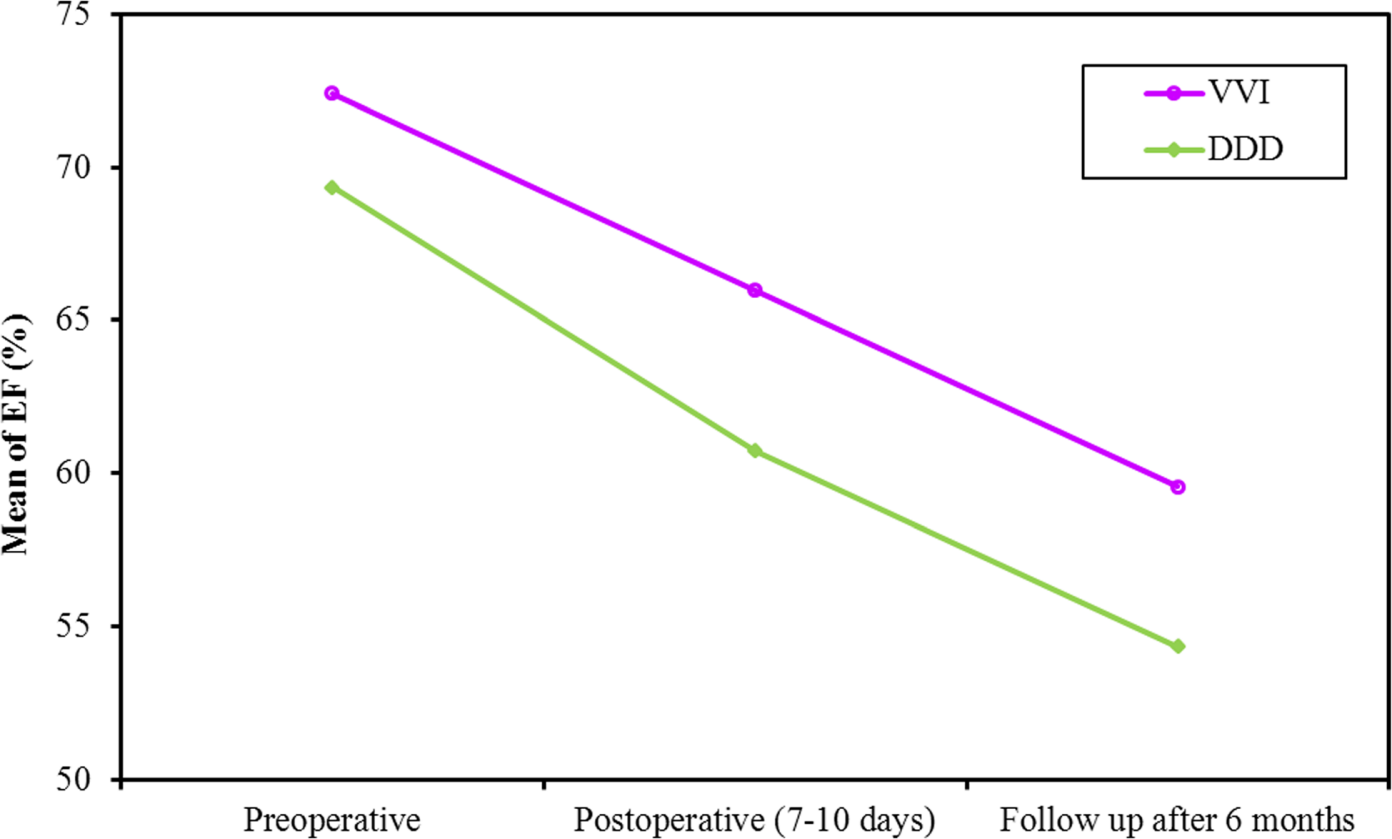
Comparison between the different periods according to EF (%) in each group

**Fig. 7:**
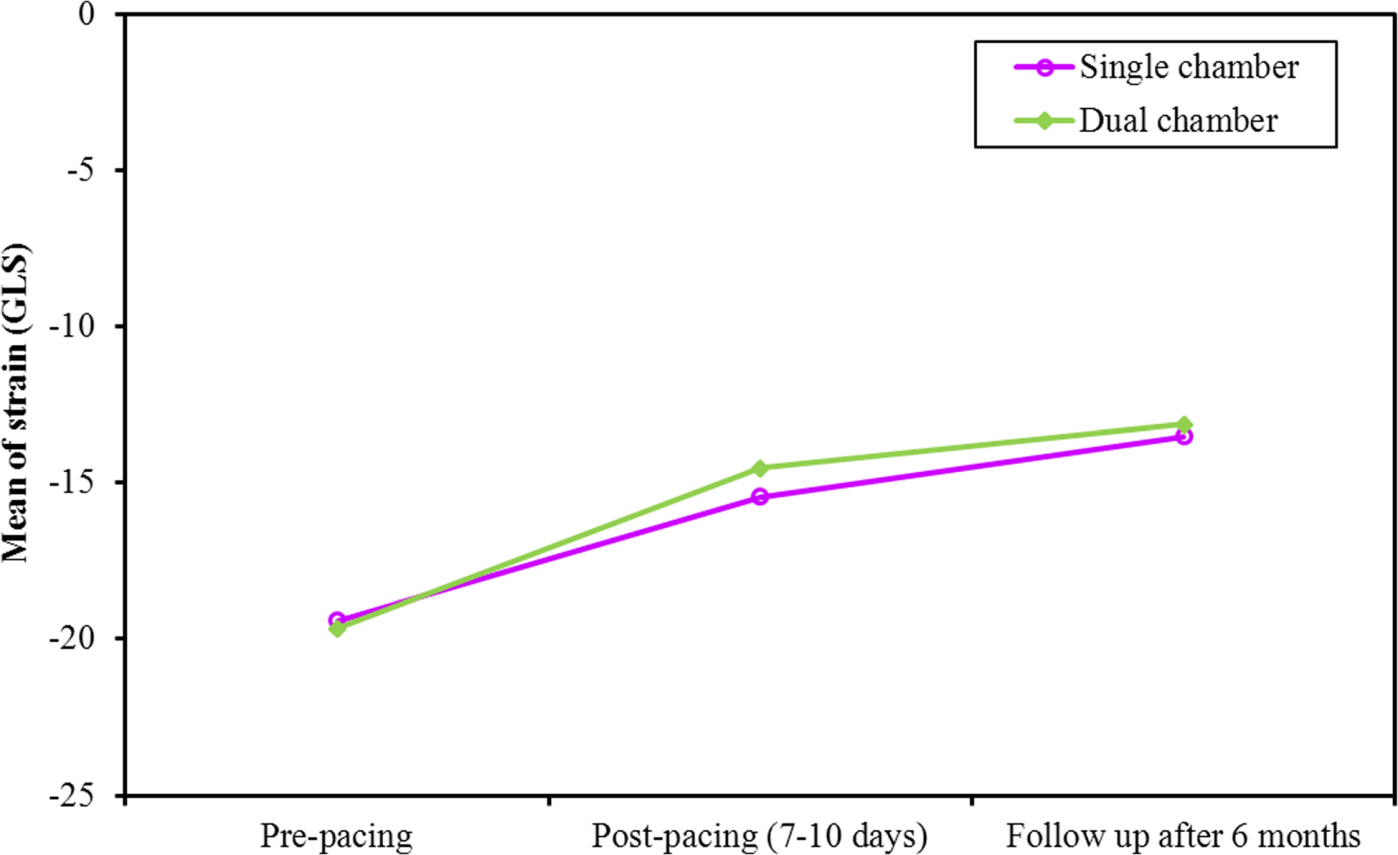
Comparison between the different periods according to strain (GLS) in each group

This reduction in SV resulted in a corresponding reduction in EF at 1 week and 6 months (*p* value < 0.001, < 0.001, respectively, Fig. 6). The reduction in EF from 1 week to 6 months was significant in single- and dual-chamber groups (*p* value = 0.001, 0.011, respectively). GLS showed a significant reduction post-pacing in both groups (*p* value <0.001, <0.001 at 1 week and 6 months, respectively). Again, the reduction in GLS from 1 week to 6 months was significant in single and dual chamber groups (Fig. 7, Table 4).

## Discussion

Our study used 3D echo and Doppler method for calculating COP. Both methods produced similar results with no significant differences. It also aimed at studying the impact of single and dual chamber pacemakers regarding their effects on left ventricular volumes and function. It was found that there was no difference between both types except for their effects on HR and COP. Dual-chamber pacemakers provided higher COP through pacing at higher HR not due to achieving AV synchrony and preserving the atrial kick. Despite the implementation of the rate response in both groups, dual-chamber pacing group managed to provide higher resting HR by tracing the patients’ intrinsic HR. Both groups showed short- and long-term detrimental effects on LV volumes and function. At 1 week, SV reduction was due to a significant drop in left ventricular EDV. Several factors contributed to the decrease in LV EDV during RV pacing:

- The drop in EDV could be a result of pacing at higher heart rates which shortened cardiac cycle duration mainly diastole leaving less time to the ventricles to fill with blood and decreasing EDV and, according to Frank-Starling law, decreasing the myocardium stretching forces leading to SV drop.
- Normal LV diastolic filling may be impaired by the loss of normal atrioventricular conduction in single-chamber pacing group with subsequent decrease in left atrial contribution to diastolic filling.
- In addition, the earlier activation of the RV may hamper LV filling that is accomplished by the shared inter-ventricular septum [18–20]. All these mechanisms resulted in a decrease in LV preload during RV apical pacing and resulted in a lower LV stroke volume.

This drop in SV had a corresponding reduction in EF at 1 week. However, the pre-pacing EF was overestimated because lower heart rates increased diastolic filling time and EDV. According to Frank-Starling law, increased myocardial stretching forces led to more efficient contraction and enhanced pre-pacing EF. Both groups showed a significant increase in COP post-pacing despite the drop in SV as it was compensated by pacing at higher heart rates.

Few published data were found on the acute effects of RV apical pacing on LV volumes and function. Our results matched with the previously published studies:

- In 2006, *Lieberman et al.* [21] compared the effects RV, LV, and biventricular (BiV) pacing in patients with preserved LV systolic function. It was found that RV apical pacing was associated with reduction in LVEF (from 51±12% to 48±14%, *P*=NS), without affecting LV dimensions.
- In 2008, *Liu et al.* [22] studied acute effects of RV apical pacing on LV function in 35 patients with sick sinus syndrome. There was a decrease in LV EDV (from 79±22 to 76±20 mL, *P*=0.07) and LVEF (from 57±8% to 54±8%, *P*=0.01) during RV pacing.
- In 2009, *Delgado et al.* [23] studied a group of 25 patients during EPS for AVNRT ablation. Left ventricular synchrony, volumes, and function were measured. There was a significant decrease in LV end-diastolic diameter and volume during RV apical pacing, whereas LV end-systolic diameter and volume did not change. Consequently, LVEF decreased significantly from 56±8% to 48±9% (*P*<0.001).

During recent years, many studies reported negative effects of RV pacing on cardiac structure and function, with detrimental clinical outcomes such as atrial fibrillation, heart failure, and death [24–26]. These changes may develop starting from the first month upto 15 years after pacemaker implantation [27].

At second follow-up, pacing led to an additional drop in EF. The main cause was pacing induced remodeling of LV structure resulting in expansion of ESV. Also, GLS dropped significantly in comparison to the measured values at 1 week. These findings signified the long-term negative impacts of RV pacing on LV function. Our study was not the first one to report a decline in LV function as a result of chronic RV pacing.

In a 6-month study of 36 patients without structural heart disease and LVEF greater than 45%, presented for permanent pacing, significant drops in LVEF were noted in 12 patients. A significant decrease in LV global longitudinal strain was noted in 23 patients [28]. Our study had a number of limitations, the most important was the small sample size. The study duration was relatively short.

The multicenter, randomized UK-PACE Trial [12] included 2021 patients presented for permanent pacing. They were followed for a mean of 4.6 years for mortality and 3 years for other cardiovascular events. It showed high annual mortality rate. No significant differences were found between the single and dual pacemakers’ patients regarding rates of atrial fibrillation, heart failure, or a composite of stroke, transient ischemic attack, or other thromboembolism.

The DAVID Trial [29] and a sub-analysis of the MADIT II [30] provided strong evidence for the negative effects of RV pacing in patients with reduced baseline LVEF. The DAVID Trial was performed in patients with reduced LVEF and receiving defibrillator for secondary prevention. It showed that single-chamber backup pacing was associated with less adverse outcomes than dual chamber pacing combined with B-blockers. The study was terminated prematurely due to reaching the composite endpoint.

In 2009, *Delgado et al.* [23] studied the acute effects of RV apical pacing on LV synchrony and mechanics. During RV apical pacing, LV contraction was more evident (from 21 ms [Q1:10, Q3:53] to 91 ms [Q1:40, Q3:204], *P*<0.001) together with an impairment in LV global longitudinal strain (from − 18.3±3.5% to − 11.8±3.6%, *P*<0.001) and in LV twist (from 12.4±3.7° to 9.7±2.6°, *P*<0.001). The study concluded that RV apical pacing induced acute LV dyssynchrony.

A recent study conducted in 2017 evaluated the value of GLS as a predictor for pacing induced LV dyssynchrony. In 93 patients followed for 5 years, cardiomyopathy developed more prevalently in dyssynchrony group (group 1: 20% vs. group 2; 3.1%, *p*<0.001). In regression analysis, lower LV global longitudinal strain was the only independent predictor for LV dyssynchrony (OR 1.252, 95% confidence interval [CI] 1.059–1.481, *p*=0.009). The study concluded that patients with lower GLS value could be at risk of cardiomyopathy and need more close monitoring [31].

All these recent trials have reached a conclusion that despite achievement of (AV) synchrony, DDD/R did not reduce pacing-related cardiomyopathy and was not superior to VVI/R mode.

## Conclusion

RV pacing led to detrimental effects on SV, COP, EF, and GLS over short- and long-term duration. Dual-chamber pacing provided higher COP than a single-chamber pacing. This was due to tracking the S.A node with pacing at higher heart rates not due to an increase in SV, preserving atrioventricular synchrony or the atrial kick. Both Doppler method and 3D echo can be used to calculate SV and COP.

## Supplementary Information

**Additional file 1.** SV by PW, 3D.

**Additional file 2.** 3D Echo.

**Additional file 3.** GLS.

## Data Availability

The datasets used and/or analyzed during the current study are available from the corresponding author on reasonable request.
