# Supplementary material for: Effects of cardiac pacemakers on left ventricular volumes and function assessed by 3D echocardiography, Doppler method, and global longitudinal strain
Source: Egypt Heart J. 2021 Feb 22;73:16. doi: 10.1186/s43044-021-00138-9 (PMC7900307; doi:10.1186/s43044-021-00138-9)
Supplement: Supplementary file 1 — Additional file 1. SV by PW, 3D. [file 43044_2021_138_MOESM1_ESM.pptx]

## Slide 1
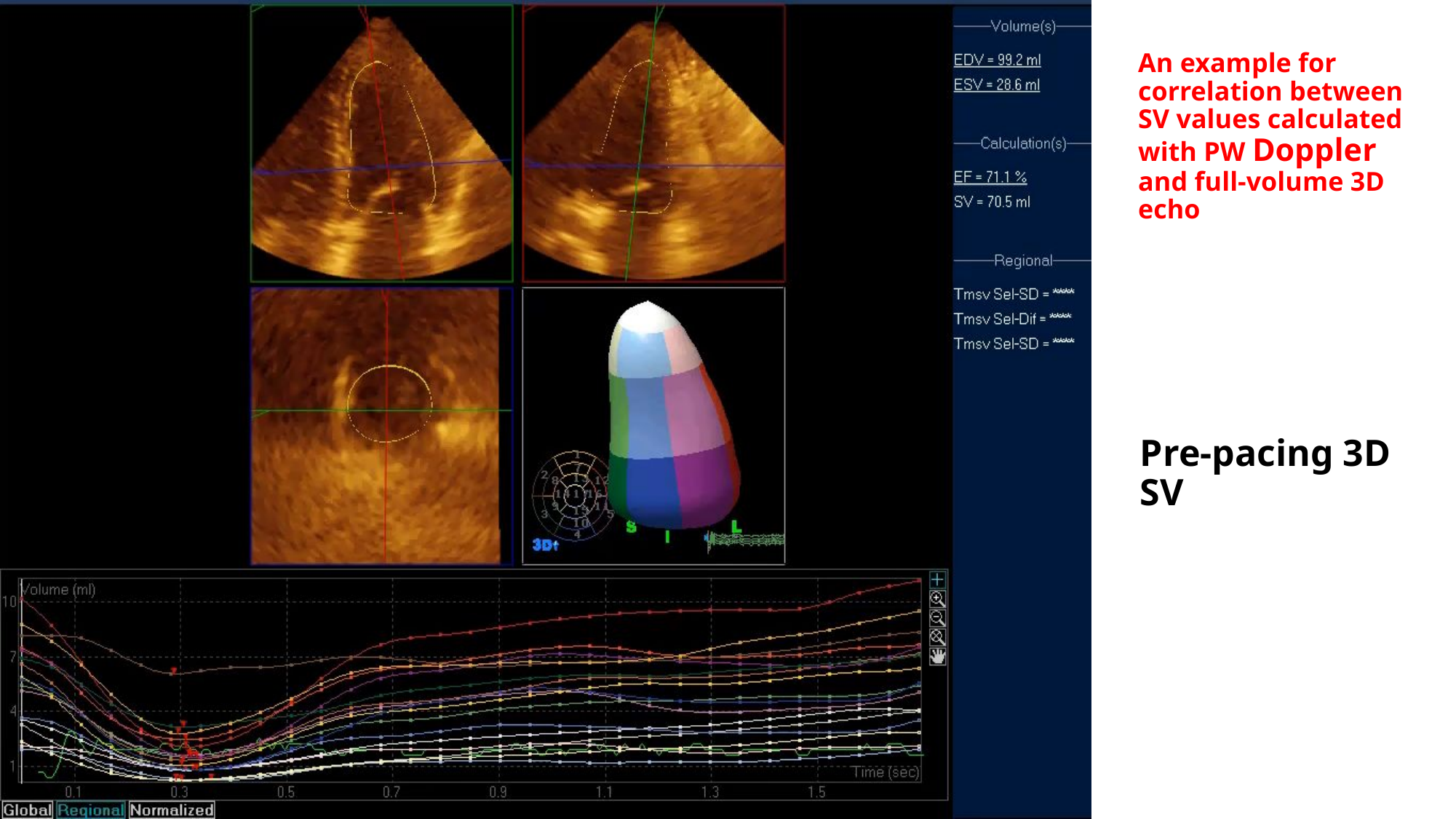

# An example for correlation between SV values calculated with PW Doppler and full-volume 3D echo
Pre-pacing 3D SV

## Slide 2
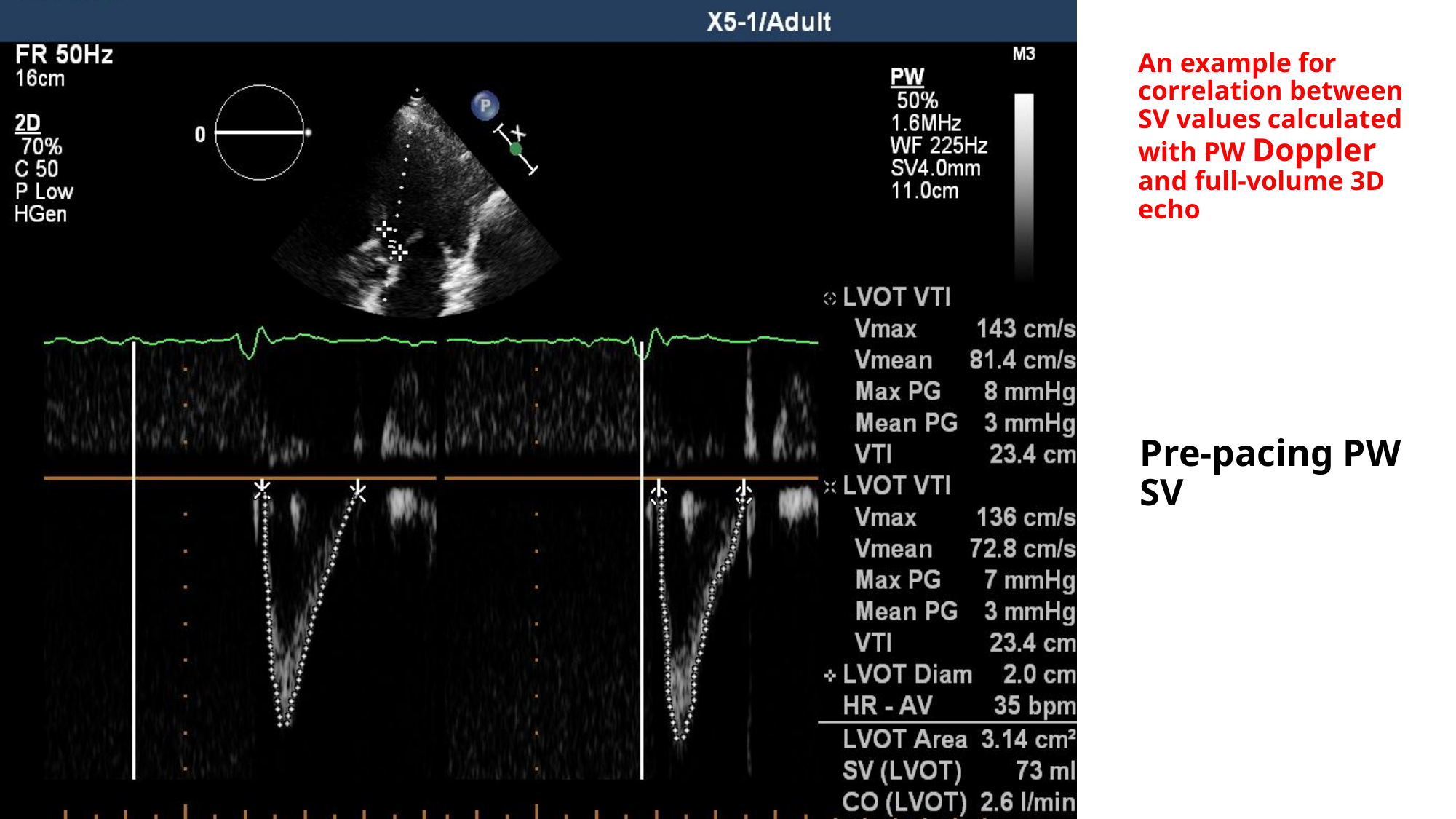

An example for correlation between SV values calculated with PW Doppler and full-volume 3D echo
Pre-pacing PW SV

## Slide 3
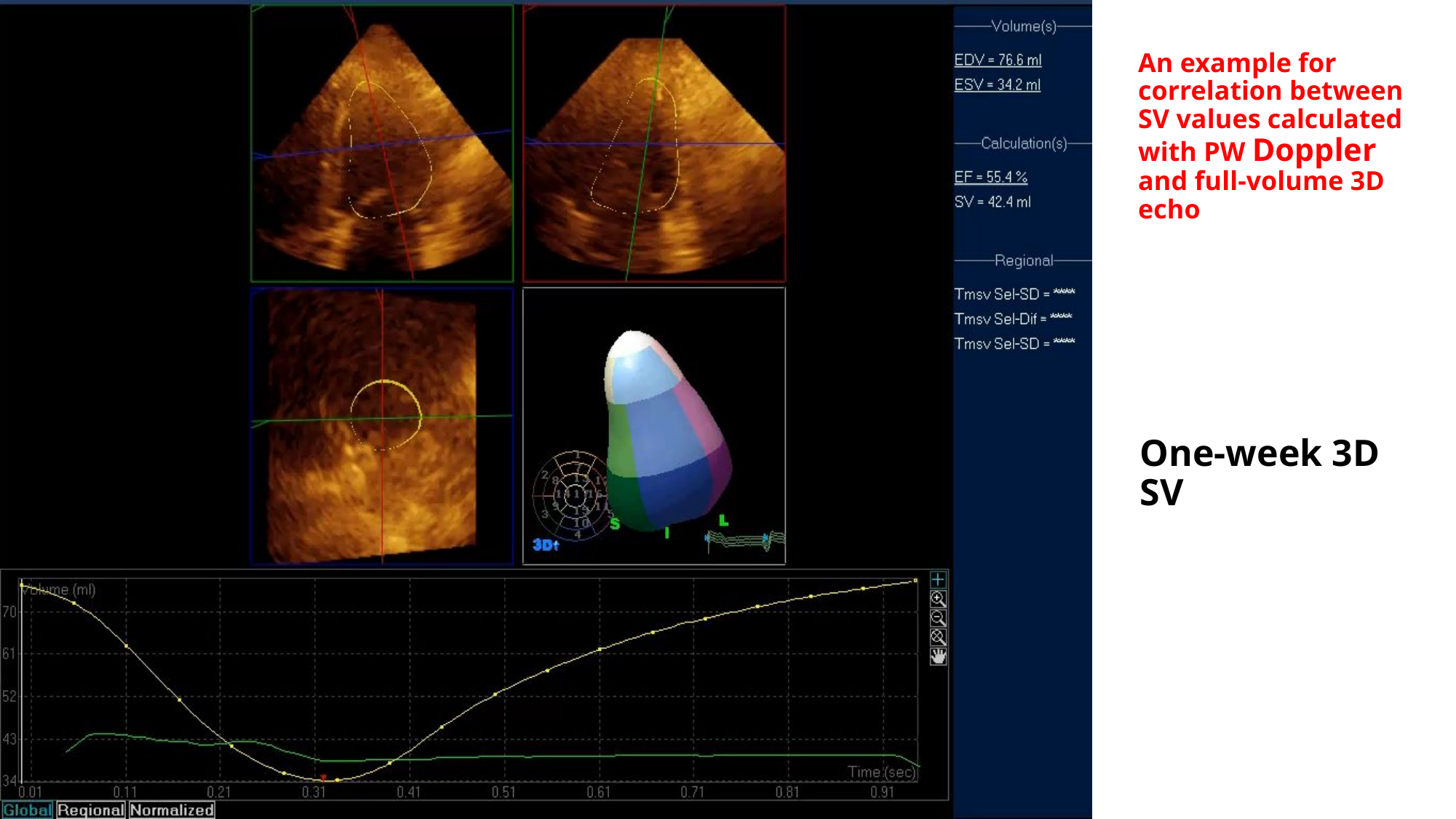

An example for correlation between SV values calculated with PW Doppler and full-volume 3D echo
One-week 3D SV

## Slide 4
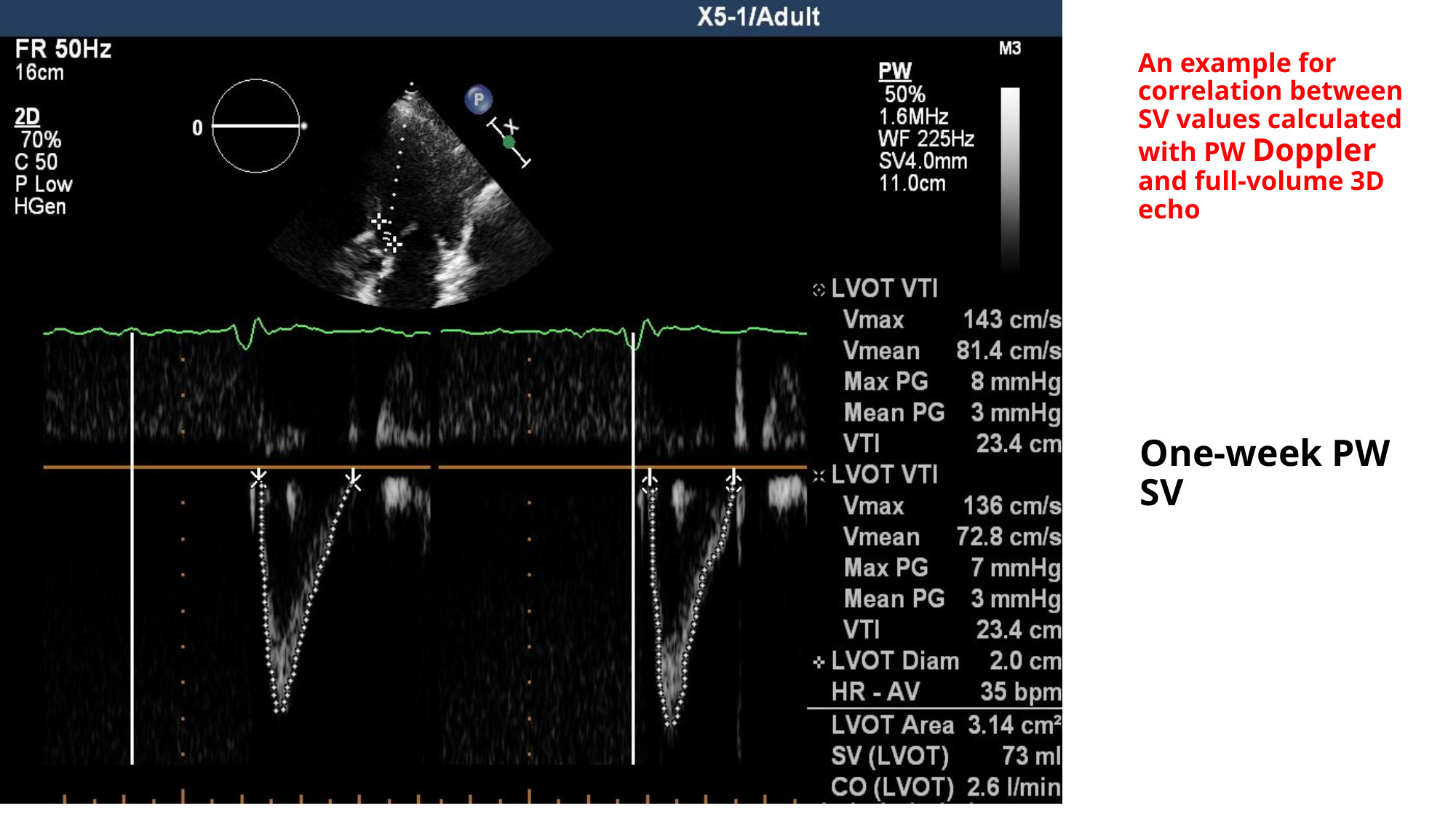

An example for correlation between SV values calculated with PW Doppler and full-volume 3D echo
One-week PW SV

## Slide 5
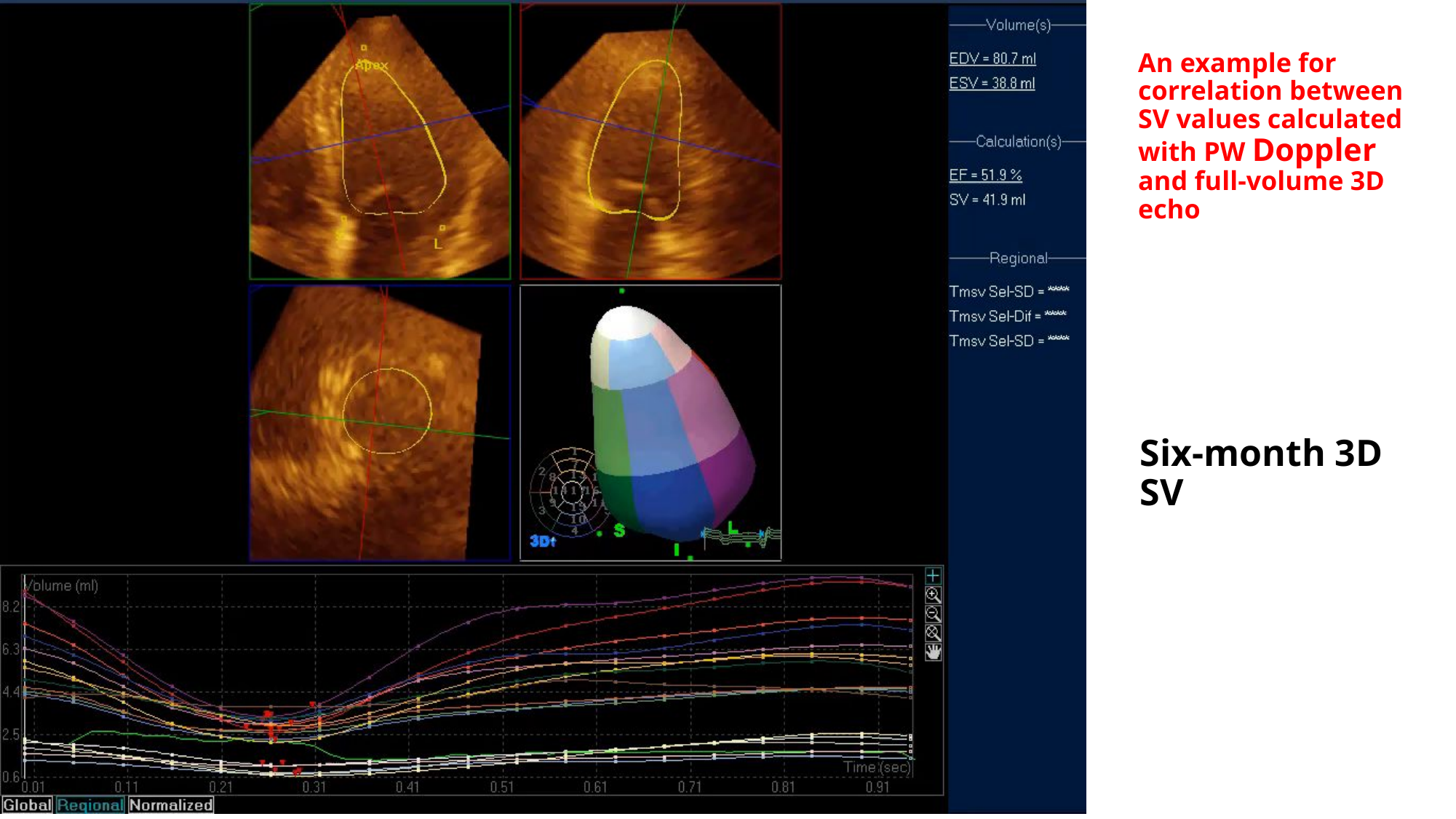

An example for correlation between SV values calculated with PW Doppler and full-volume 3D echo
Six-month 3D SV

## Slide 6
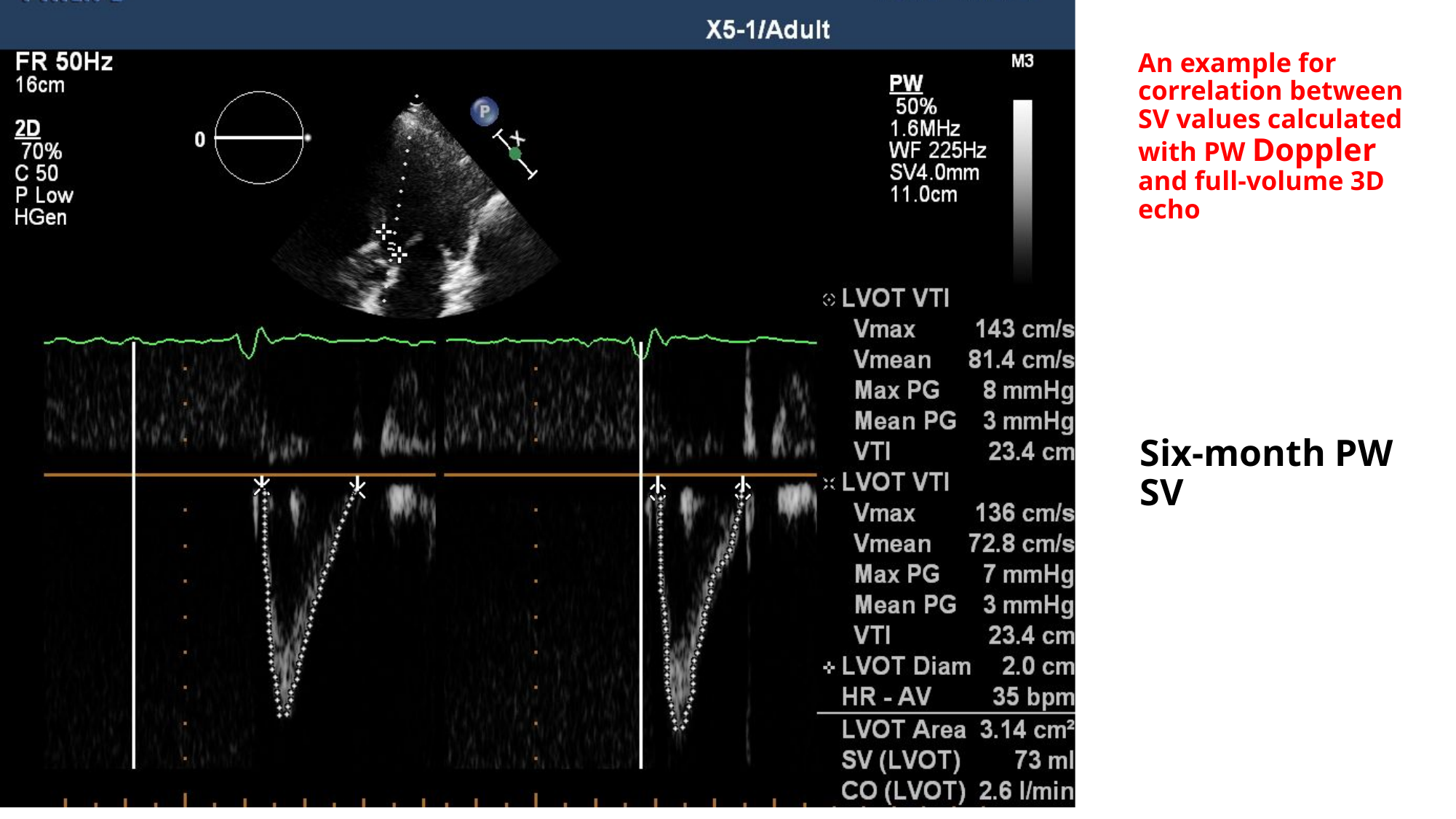

An example for correlation between SV values calculated with PW Doppler and full-volume 3D echo
Six-month PW SV

## Slide 7
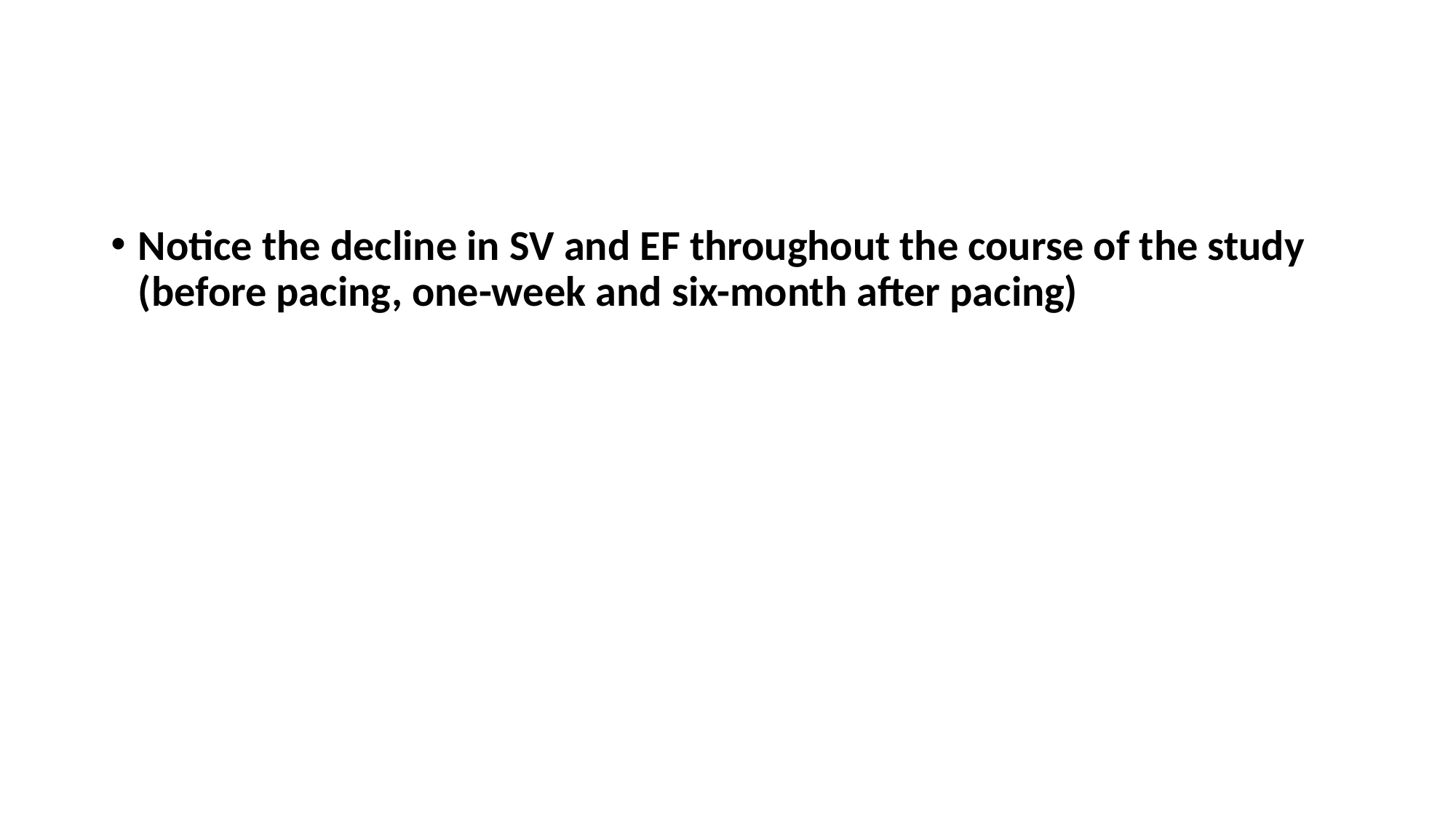

#
Notice the decline in SV and EF throughout the course of the study (before pacing, one-week and six-month after pacing)
