# Supplementary figures and images for: Effects of cardiac pacemakers on left ventricular volumes and function assessed by 3D echocardiography, Doppler method, and global longitudinal strain
Source: Egypt Heart J. 2021 Feb 22;73:16. doi: 10.1186/s43044-021-00138-9 (PMC7900307; doi:10.1186/s43044-021-00138-9)

## Slide 1
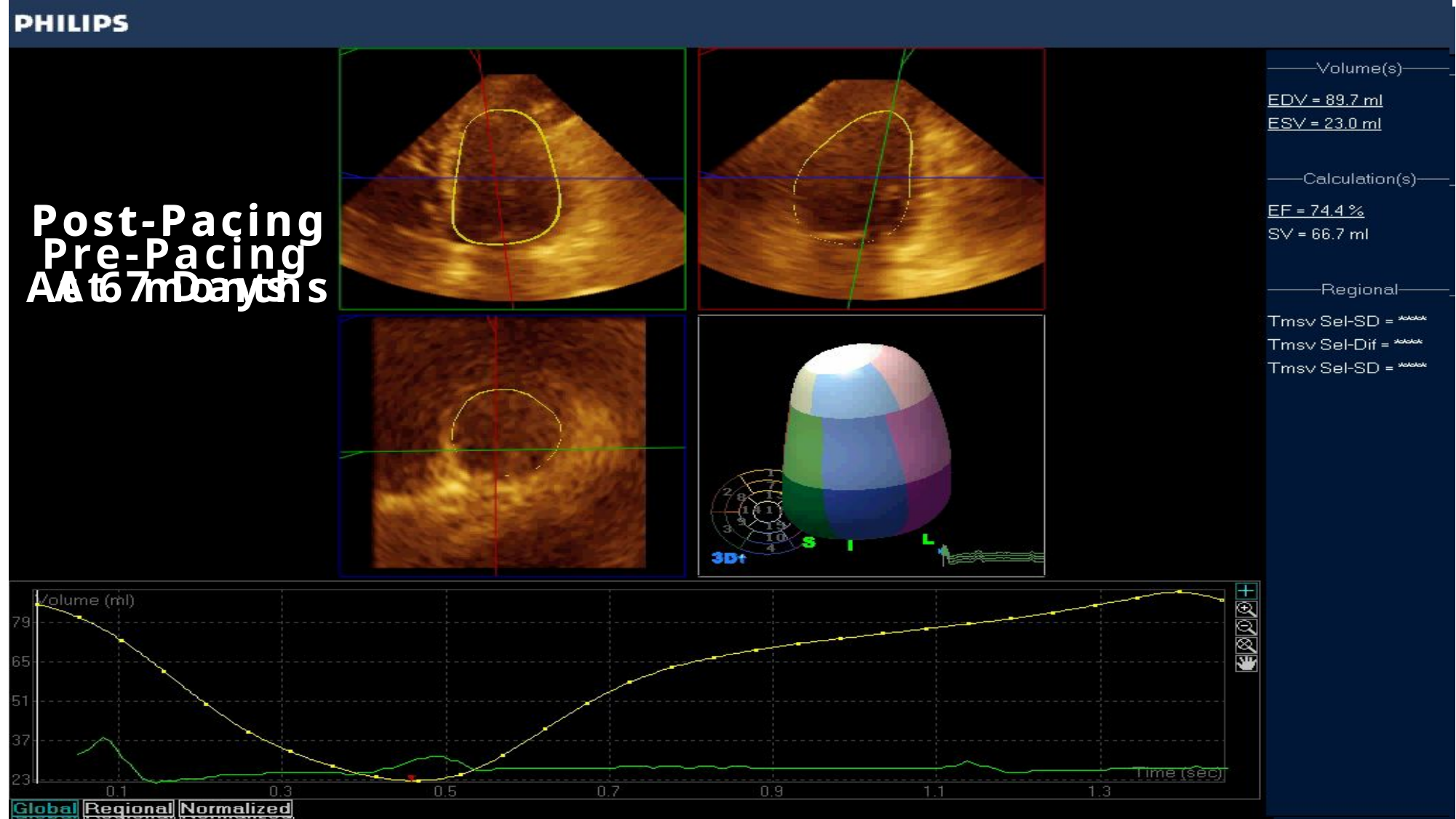

Post-Pacing
At 7 Days
Post-Pacing
At 6 months
Pre-Pacing

## Slide 2
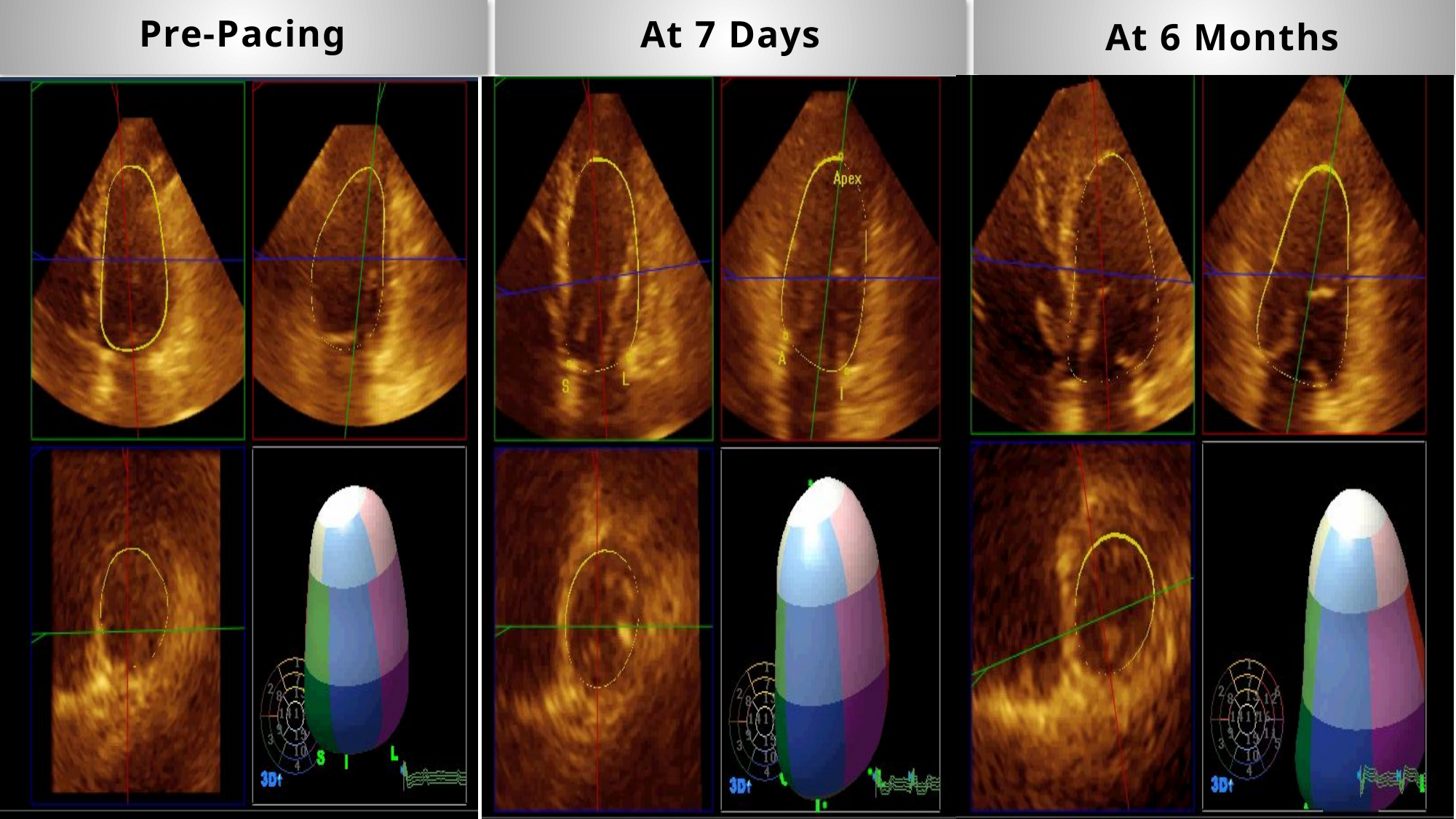

Pre-Pacing
At 7 Days
At 6 Months

Supplement: Supplementary file 2 — Additional file 2. 3D Echo. [file 43044_2021_138_MOESM2_ESM.pptx]

## Slide 1
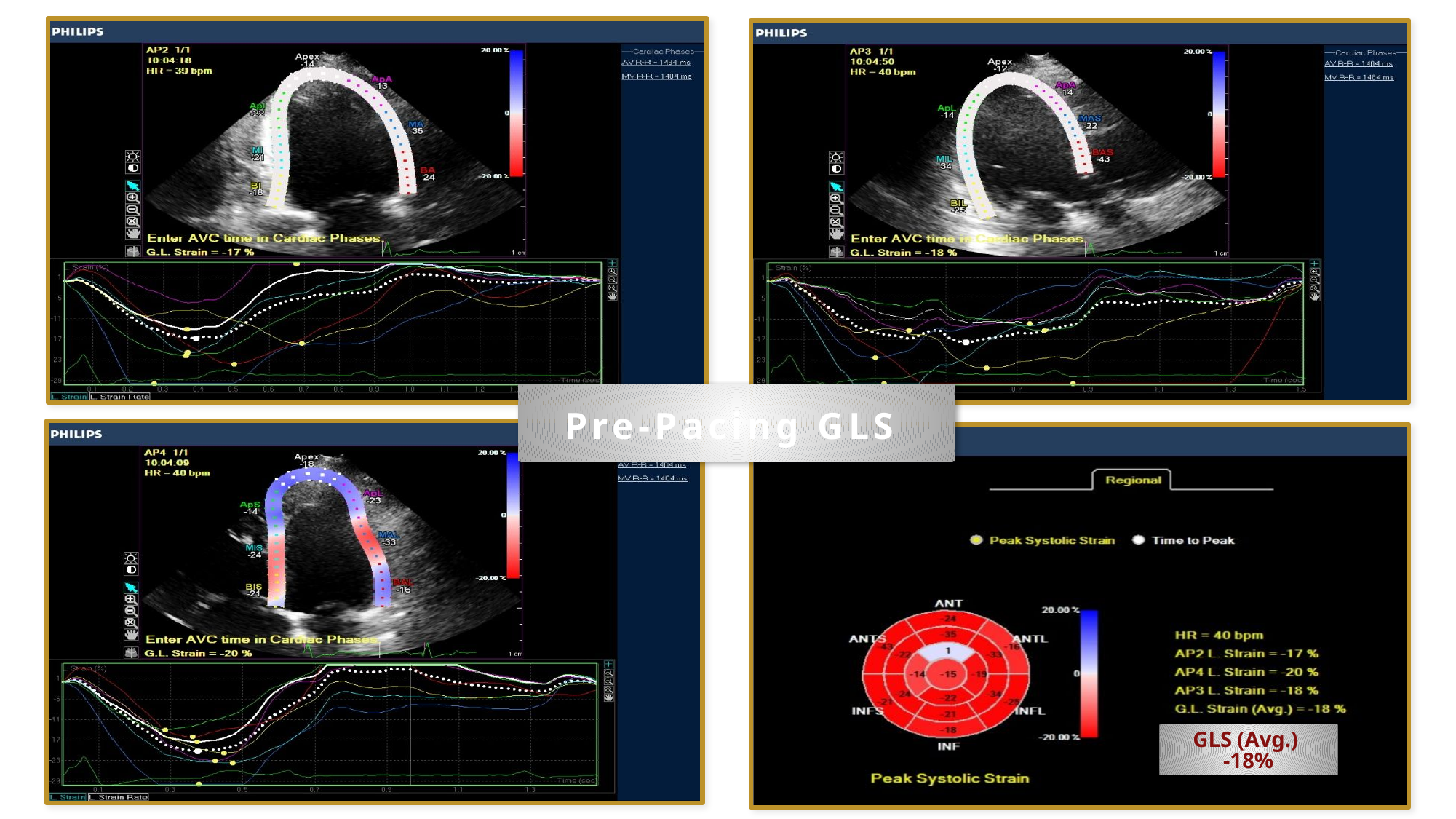

Pre-Pacing GLS
GLS (Avg.)
-18%

## Slide 2
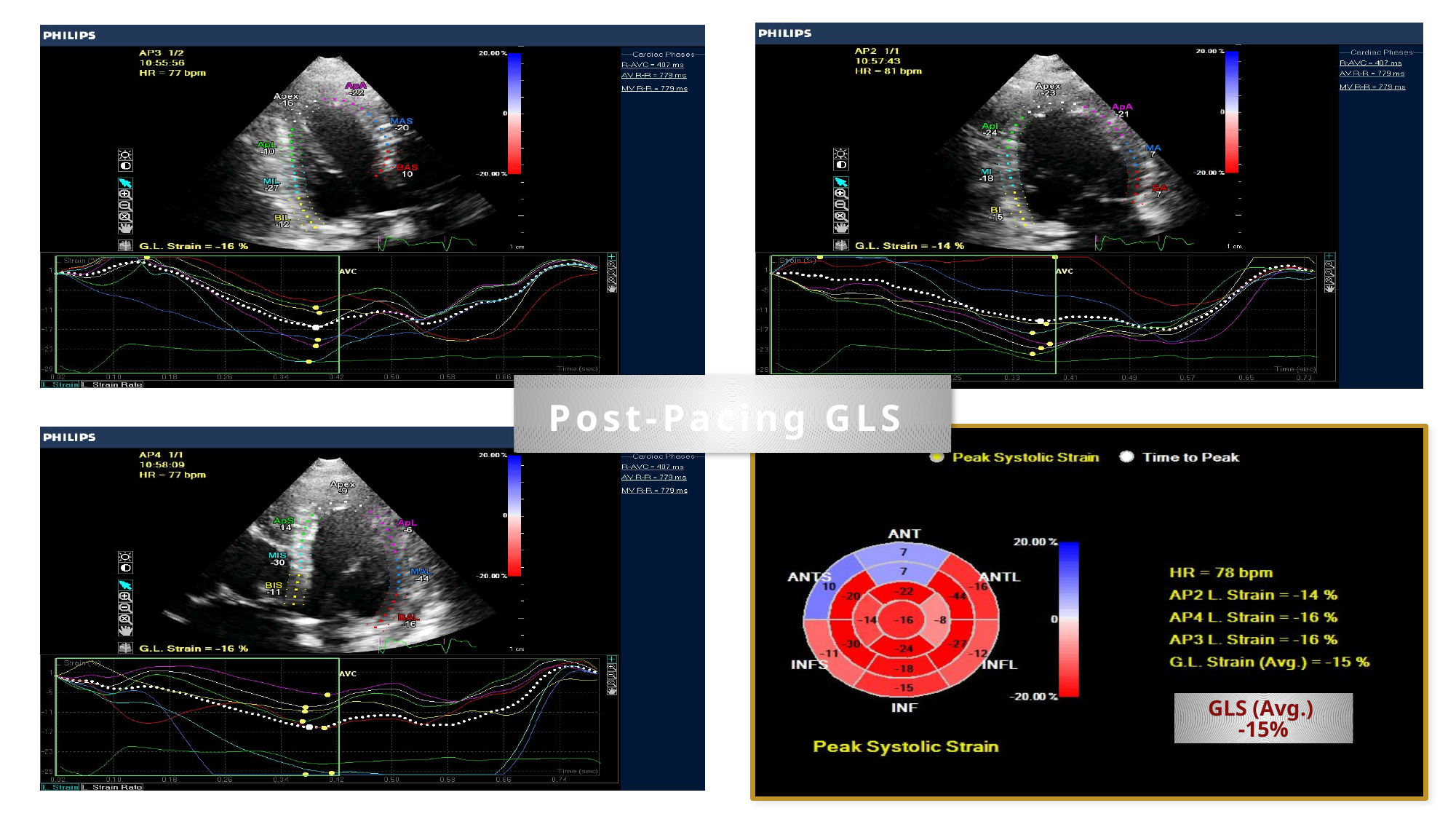

Post-Pacing GLS
GLS (Avg.)
-15%

Supplement: Supplementary file 3 — Additional file 3. GLS. [file 43044_2021_138_MOESM3_ESM.pptx]
